# Managing bleeds on anticoagulant therapy: a practical guide for clinicians

**DOI:** 10.1093/ehjacc/zuag035

**Published:** 2026-03-12

**Authors:** Mattia Galli, Beatrice Simeone, Jurrien ten Berg, Davide Capodanno, Marco Valgimigli, Sebastiano Sciarretta, Ernesto Greco, Adnan Kastrati, Gilles Montalescot, C Michael Gibson, Diana A Gorog, Roxana Mehran, Dominick J Angiolillo

**Affiliations:** Department of Medical-Surgical Sciences and Biotechnologies, Sapienza University of Rome, Corso della Repubblica, 79, Latina 04100, Italy; Department of Cardiology, Maria Cecilia Hospital, GVM Care & Research, Cotignola 48033, Italy; Department of Medical-Surgical Sciences and Biotechnologies, Sapienza University of Rome, Corso della Repubblica, 79, Latina 04100, Italy; Department of Cardiology, St.Antonius Hospital, Nieuwegein, the Netherlands; Department of Cardiology, Maastricht Medical Centre, Maastricht, the Netherlands; Division of Cardiology, Azienda Ospedaliero-Universitaria Policlinico Rodolico—San Marco, University of Catania, Catania, Italy; Cardiocentro Ticino Institute, Ente Ospedaliero Cantonale, University of Italian Switzerland, Lugano, Switzerland; Department of Cardiology, Bern University Hospital, University of Bern, Bern, Switzerland; Department of Medical-Surgical Sciences and Biotechnologies, Sapienza University of Rome, Corso della Repubblica, 79, Latina 04100, Italy; Department of Angiocardioneurology, IRCCS NeuroMed, Pozzilli, Italy; Department of Health and Life Sciences, European University of Rome, Rome, Italy; Klinik für Herz- und Kreislauferkrankungen, TUM Klinikum Deutsches Herzzentrum, Technische Universität München, Munich, Germany; Deutsches Zentrum für Herz- und Kreislauf-Forschung (DZHK) e.V. (German Centre for Cardiovascular Research), Partner Site Munich Heart Alliance, Munich, Germany; ACTION Study Group, INSERM UMRS1166, Sorbonne Université, Hôpital Pitié-Salpêtrière (AP-HP), Paris, France; Baim Institute for Clinical Research, Harvard Medical School, Harvard University, Boston, MA, USA; Department of Cardiology, University of Hertfordshire, Hatfield, Hertfordshire, UK; National Heart and Lung Institute, Imperial College, London, UK; Mount Sinai Fuster Heart Hospital, Icahn School of Medicine at Mount Sinai, NewYork, NY, USA; Division of Cardiology, University of Florida College of Medicine, 655 West 8th Street—Jacksonville, Jacksonville, FL 32209, USA

**Keywords:** Bleeding, Anticoagulation, Reversal strategies, Anticoagulant therapy, Haemostasis, Risk stratification

## Abstract

Bleeding is a prevalent and frequently serious complication of anticoagulant therapy. The use of oral anticoagulants to prevent thrombotic events in common cardiovascular conditions, such as atrial fibrillation, exposes patients to an increased risk of bleeding which is associated with morbidity and mortality. The consequences of bleeding extend well beyond the acute event, affecting treatment adherence, long-term management decisions, and increasing the risk of ischaemic events when anticoagulation is discontinued. Optimizing the balance between thrombotic and bleeding risk requires expertise in risk stratification, preventive strategies, acute management, and the appropriate timing of anticoagulation resumption after haemostasis. This review summarizes the epidemiology and clinical impact of bleeding in patients receiving oral anticoagulant therapy and provides contemporary, patient-centred flowcharts for acute management and safe resumption of treatment. Future efforts should focus on refining risk prediction, individualizing anticoagulant regimens, and improving access to targeted reversal agents to further improve patient outcomes.

## Introduction

Oral anticoagulants (OACs) are the cornerstone of prevention and treatment of thrombotic events across a wide spectrum of cardiovascular conditions, including atrial fibrillation (AF), venous thromboembolism (VTE), and mechanical heart valve prostheses.^1,2^ Given increasing life expectancy and the growing prevalence of multimorbidity, the number of patients exposed to long-term oral OAC therapy is steadily rising, with a parallel increase in treatment-related adverse events, particularly bleeding.^3^

Despite the well-established efficacy of OACs in reducing thromboembolic complications, bleeding remains the most frequent complication of anticoagulant therapy, exerting a direct impact on morbidity, mortality, and premature treatment discontinuation.^3,4^ Importantly, the prognostic relevance of bleeding extends beyond the acute event, as haemorrhagic complications frequently trigger interruption or permanent withdrawal of anticoagulation, thereby exposing patients to an excess risk of ischaemic stroke, systemic embolism, myocardial infarction (MI), and death.^4^ While major bleeding events, particularly intracranial and gastrointestinal (GI) haemorrhages, are consistently associated with substantial short- and long-term mortality,^5,6^ growing evidence indicates that also minor bleeding should not be regarded as a benign complication. Indeed, non-major bleeding episodes often lead to clinically relevant therapeutic modifications, including dose reduction or abrupt discontinuation of anticoagulant therapy, which may translate into an increased risk of subsequent ischaemic events.^3,7^

In this context, bleeding has emerged as a central determinant of prognosis in anticoagulated patients, often outweighing thrombotic complications in terms of clinical impact.^5^ This paradigm shift has fuelled increasing interest in structured bleeding prevention, standardized severity assessment, and evidence-based management pathways aimed at minimizing haemorrhagic risk without compromising antithrombotic efficacy.^3,4^

In this review, we provide a practical and evidence-based framework for clinicians managing bleeding in patients treated with OACs. We discuss bleeding prevention strategies, structured assessment of severity, management of minor and major bleeding events, appropriate use of reversal agents, and a pragmatic flow chart designed to support clinical decision-making in acute care settings.

## Epidemiology and prognostic implications

Bleeding remains one of the most frequent and clinically relevant adverse events in patients treated with OACs, with an incidence rate strongly influenced by drug class, concomitant medications, comorbidity burden, treatment duration, and intensity.^3,4^

Importantly, bleeding risk is not constant over time after initiation of oral anticoagulation.^8^

Observational studies and *post hoc* analyses of randomized trials suggest that bleeding risk is highest in the early phase of treatment, particularly during the first weeks to months after initiation. Thereafter, bleeding rates tend to stabilize, although they remain clinically relevant during long-term follow-up, especially in older patients and those with multiple comorbidities.^8–10^

Across contemporary cardiovascular cohorts, major bleeding occurs in approximately 1–3% per year, with higher rates observed in elderly and frail populations.^11,12^ The introduction of direct oral anticoagulants (DOACs) has fundamentally reshaped the bleeding landscape. Compared with vitamin K antagonists (VKAs), DOACs provide a ∼50% reduction in intracranial haemorrhage (ICH) and a significant decrease in fatal bleeding.^13^ In randomized trials of AF, VKAs are consistently associated with a major bleeding rate of about 3% annually, whereas DOACs reduce this risk to approximately 2%.^13^ This overall safety advantage may come at the expense of a higher incidence of GI bleeding with certain agents and doses—particularly rivaroxaban and high-dose dabigatran—while apixaban demonstrates the most favourable GI safety profile in network meta-analyses.^14^ Combination antithrombotic therapy (i.e. adding antiplatelet therapy to OAC) further amplifies bleeding risk.^15,16^

In this context, the Academic Research Consortium for High Bleeding Risk (ARC-HBR) classification explicitly recognizes chronic OAC use as a major criterion for high bleeding risk (HBR), reflecting the intrinsic haemorrhagic liability associated with anticoagulated patients undergoing PCI.^3,4,6,17^ Importantly, OAC-related bleeding risk is further modulated by additional clinical factors, including advanced age, prior bleeding, chronic kidney disease (CKD), anaemia, and concomitant use of non-steroidal anti-inflammatory drugs (NSAIDs) or corticosteroids, which frequently cluster in this population and markedly amplify haemorrhagic risk across clinical settings.^3,4,18^ Consistent with this multifactorial paradigm, evidence from the derivation and validation of the PRECISE-HBR score indicates that, although OAC use is a major contributor to bleeding risk after PCI, it does not uniformly translate into a HBR phenotype, with a non-negligible proportion of OAC-treated patients remaining below the ARC-HBR threshold of ≥4% 1-year BARC 3 or 5 bleeding.^19^

The prognostic impact of bleeding is substantial and extends well beyond the acute episode. Major bleeding—especially intracranial, retroperitoneal, pericardial, or severe GI events—is independently associated with increased short- and long-term mortality, often exceeding that of MI.^20,21^ In large AF cohorts, 30-day mortality after major bleeding surpasses 15%, and 1-year mortality exceeds 25%.^7^ Even non-major bleeding may carry meaningful clinical impact, as it frequently leads to therapy interruption, use of antidotes or blood transfusions that may expose patients to an elevated risk of thromboembolic complications.^7,22^

Importantly, bleeding-related discontinuation of anticoagulant therapy is a major driver of subsequent thrombotic events and mortality, underscoring the need for structured reassessment and timely reinitiation once haemostasis is achieved.^4^ In contemporary practice, bleeding-related hospitalizations are increasing, largely due to broader OAC use in ageing, multimorbid populations and the persistent use of combination regimens in anticoagulated patients.^23^

Taken together, bleeding complications among patients on OAC are not incidental events but strong prognostic determinants. Recognizing bleeding as a central driver of morbidity and mortality highlights the need for proactive prevention, systematic assessment, and structured management strategies across the whole anticoagulation pathway, from initiation to long-term follow-up.

## Prevention of bleeds on anticoagulant therapy

Optimal prevention of bleeding events involves: (i) individualized bleeding risk stratification with periodic reassessment over time; (ii) recognition that bleeding and thrombotic risks may evolve and require optimal balancing; (iii) careful selection of the OAC class and dose tailored to individual patient characteristics and the clinical scenario; (iv) consideration of concomitant medications; and (v) implementation of specific bleeding-avoidance strategies.

Accurate identification of HBR patients is essential to enable timely implementation of bleeding-avoidance strategies and to ensure consistent risk stratification across clinical trials. Among patients undergoing PCI, multiple bleeding risk scores have been developed, differing by clinical setting, target population, outcome definition, and methodological approach. Those supported by more robust evidence and most widely used include the PRECISE-DAPT score, the ARC-HBR classification, and the more recent PRECISE-HBR score.^17,19,24,25^

Although these tools were primarily derived and validated in antiplatelet-treated populations, they may provide a pragmatic framework for bleeding risk stratification in contemporary PCI practice, including patients with an indication for OAC. Beyond formal risk scores, bleeding risk is further shaped by the cumulative burden and interaction of multiple clinical and procedural factors. While many of these variables are incorporated within existing scores, their individual presence, severity, and clustering, such as advanced age, CKD, liver disease, prior bleeding, anaemia, thrombocytopenia, malignancy, non-radial access, intensive periprocedural antithrombotic therapy (and, in selected settings, East Asian ethnicity), remain clinically relevant and should be integrated into a comprehensive patient-level assessment.^26–30^

Among AF patients treated with OAC, several validated clinical scores help estimate long-term bleeding vulnerability in patients receiving OACs or antiplatelet therapy. Tools such as HAS-BLED,^31^ ORBIT,^32^ ATRIA,^33^ GARFIELD-AF,^34^ HEMORR_2_HAGES, ABC-Bleeding,^35^ and the recently proposed DOAC score^36^ are summarized in *Table 1* and support individualized preventive strategies, although they should not replace clinical judgment in acute settings.

**Table 1:** Validated bleeding risk prediction models

Score	Main variables	Intended population	Predictive focus	Interpretation	Key notes
HAS-BLED^31^	Hypertension, Abnormal renal/liver function, stroke, bleeding history, labile INR, elderly (>65 years), drugs/alcohol	AF on OAC (VKA or DOAC)	Major bleeding	≥3 = high risk; 1–2 = intermediate; 0 = low	Widely validated; emphasizes modifiable risk factors; recommended by ESC to identify correctable risks, not to exclude OAC.
ORBIT^32^	Age ≥75, anaemia (low Hb/Hct), bleeding history, kidney function, antiplatelet therapy	AF on OAC (including DOACs)	Major bleeding	≥4 = high risk; 2–3 = intermediate; 0–1 = low	Developed from DOAC-treated cohorts; robust external validation; useful when HAS-BLED is inconclusive.
ATRIA^33^	Anaemia, severe renal disease, age ≥75, hypertension, prior bleeding	AF on OAC	Major bleeding	≥5 = high risk; 4 = moderate; ≤3 = low	Older model derived from warfarin data; underestimates bleeding in elderly and DOAC-treated patients.
GARFIELD-AF^34^	Age, sex, CAD/PAD, CKD, hypertension, diabetes, smoking, prior bleeding, alcohol	AF (VKA or DOAC)	Major bleeding and mortality	Continuous model; higher scores = greater risk	Integrates bleeding and mortality; dynamic recalibration with registry data; suitable for longitudinal risk reassessment.
HEMORR_2_HAGES^37^	Hepatic/renal disease, ethanol abuse, malignancy, older age, reduced platelet/function, hypertension, anaemia, genetic factors, excessive fall risk, stroke	AF on OAC (VKA)	Major bleeding	≥5 = high risk	Historic model; less used today; includes fall risk and genetic variables (e.g. *CYP2C9*).
ABC-Bleeding^35^	Age, biomarkers (GDF-15, hs-TnT, Hb), clinical history (prior bleeding)	AF on OAC (VKA or DOAC)	Major bleeding	Continuous model; upper tertile = high risk	Biomarker-based; highest predictive accuracy; recommended when biomarkers are available.
DOAC Score^36^	Age ≥75, anaemia, CKD, prior bleeding, antiplatelet therapy	AF on DOAC	Major bleeding	≥2 = high risk; 1 = intermediate; 0 = low	Specifically designed for DOAC-treated patients; simple and validated; may outperform HAS-BLED in this population.
PRECISE-HBR^19^	Age, haemoglobin, white blood cell count, eGFR, prior bleeding, oral anticoagulation, ARC-HBR criteria	PCI patients, including those on OAC	1-year major bleeding (BARC 3–5)	≥23 = high bleeding risk (≥4% at 1 year)	Integrates PRECISE-DAPT and ARC-HBR; improves bleeding discrimination vs. PRECISE-DAPT and ARC-HBR alone; validated in contemporary PCI cohorts including OAC-treated patients

AF, atrial fibrillation; ARC-HBR, Academic Research Consortium–High Bleeding Risk; BARC, Bleeding Academic Research Consortium; BMI, body mass index; BP, blood pressure; CAD, coronary artery disease; CKD, chronic kidney disease; DOAC, direct oral anticoagulant; eGFR, estimated glomerular filtration rate; GDF-15, growth differentiation factor-15; Hb, haemoglobin; hs-TnT, high-sensitivity cardiac troponin T; INR, international normalized ratio; OAC, oral anticoagulant; PAD, peripheral artery disease; PCI, percutaneous coronary intervention; PRECISE-HBR, Predicting Bleeding Complications in Patients Undergoing Stent Implantation and Subsequent Dual Antiplatelet Therapy–High Bleeding Risk; VKA, vitamin K antagonist; WBC, white blood cell count.

In patients on long-term OAC, a key factor to prevent major haemorrhagic events lies in judicious modulation of concomitant antiplatelet therapy. Among patients undergoing PCI, approximately 10–15% have an indication for chronic oral anticoagulation, most commonly AF, placing them in a particularly vulnerable clinical setting in which exposure to combined antithrombotic regimens is frequent and haemorrhagic complications are prognostically relevant.^18^ Within this high-risk population, the combination of OAC with DAPT—historically referred to as triple antithrombotic therapy (TAT)—has consistently been associated with a two- to three-fold increase in major bleeding compared with OAC-based dual regimens or OAC alone, as shown in large observational cohorts and randomized studies.^15,38^

This paradigm was first established in the WOEST trial, which demonstrated that omission of aspirin at the time of PCI, resulting in the use of dual antithrombotic therapy with OAC plus clopidogrel significantly reduced bleeding complications compared with long-term (12 months) TAT with VKA, aspirin and clopidogrel, without an increase in thrombotic events.^39^ Subsequent RCTs and meta-analyses have confirmed that early aspirin discontinuation after PCI in anticoagulated patients significantly reduces bleeding risk without a clear increase in ischaemic outcomes.^40^ However, an increase in thrombotic events is possible in patients at high thrombotic risk, such as those presenting with ACS or undergoing complex PCI, in whom TAT for up to 1 month may be considered.^15,41,42^ These observations underscore the need for careful patient-level risk stratification and individualized antithrombotic strategies rather than a uniform de-escalation approach. Accordingly, contemporary practice supports reducing the association of OAC with antiplatelet agents as much as possible, while the combination of OAC with two antiplatelet drugs should be avoided whenever feasible and, in patients presenting with ACS, limited to the shortest duration required, up to 7 days and generally not exceeding one month, in line with the temporal dissociation between ischaemic and bleeding risk and the principles of antithrombotic de-escalation.^18,43,44^

For long term treatment of PCI patients (i.e. 6–12 months after PCI) or high-risk chronic coronary syndrome patients who require OAC, solid evidence confirms that the addition of aspirin to OACs is associated with a marked increase in both minor and major, without ischaemic benefit.^45^ Therefore, OAC alone (i.e. without antiplatelet therapy) is recommended in these patients.^46,47^ Strategies for bleeding prevention should also address modifiable bleeding co-triggers that frequently coexist in anticoagulated populations. Concomitant use of NSAIDs significantly increases GI bleeding risk in patients on OAC, particularly in those with prior MI,^48^ while selective serotonin reuptake inhibitors (SSRI) have been independently associated with higher rates of major and clinically relevant non-major bleeding when combined with OACs.^49^ Systemic corticosteroids further amplify GI bleeding risk, as consistently shown in meta-analytic data.^50^ Careful medication reconciliation and targeted patient counselling therefore remain integral components of bleeding prevention in patients treated with OAC.

Gastroprotection represents one of the most effective and evidence-based strategies. Observational cohorts and meta-analyses have extended the benefit to anticoagulated populations, showing a consistent reduction in upper GI bleeding across all OAC classes.^51^ Current ESC guidelines recommend routine PPI therapy in high-risk patients treated with DAPT or OAC.^46,52^

Adherence to on-label dosing is another pillar of bleeding prevention. Inappropriate DOAC underdosing—often driven by fear of bleeding—fails to reduce haemorrhagic events and increases stroke and mortality risk.^53,54^ Patient-level meta-analysis confirms that label-adherent DOAC dosing provides the most favourable balance between efficacy and safety across the spectrum of renal function.^55^ Regular renal re-evaluation and avoidance of off-label dose reduction are therefore central to safe long-term care.^56^

Finally, standardized peri-procedural management is essential to minimize iatrogenic bleeding. The PAUSE trial established that simple, dialysis-adjusted DOAC interruption protocols without heparin bridging result in low rates of major bleeding and thromboembolism.^57^ For VKA-treated patients, the BRIDGE trial demonstrated that low-molecular-weight heparin (LMWH) bridging increases major bleeding without reducing thromboembolic events, confirming that bridging should be reserved for exceptional high-risk indications.^58^ In agreement with these data, the PERIOP2 randomized trial showed no significant differences in either thromboembolic or major bleeding events with postoperative LMWH bridging compared with placebo.^59^

## Assessing bleeding severity

### Defining bleeding severity

Accurate assessment of bleeding severity is pivotal to guiding early management, determining the need for escalation, and supporting the appropriate use of reversal agents. Multiple societies and academic groups have proposed structured bleeding definitions, including TIMI,^60,61^ GUSTO, ISTH,^62–64^ ACUITY,^65^ and the Bleeding Academic Research Consortium (BARC),^6^ that integrate clinical impact, anatomic site, and laboratory parameters. Among these, BARC has become the most widely adopted framework in trials and guidelines, offering a pragmatic spectrum from minor events to fatal bleeding.^6^

A concise comparison of major definitions and their clinical implications is provided in *Table 2*.

**Table 2:** Bleeding classification systems in cardiovascular medicine

Classification system	Definition criteria	Clinical correlates	Prognostic impact
TIMI (Thrombolysis in Myocardial Infarction; non CABG)^60,61^	**Major:** Any intracranial haemorrhage or a Hb decrease ≥5 g/dL. **Minor:** Hb fall of 3–5 g/dL. **Minimal:** Any overt bleeding event not meeting previous criteria.	Primarily laboratory-based classification relying on Hb drop, regardless of clinical impact.	Major TIMI bleeds are associated with increased 30-day and 1-year mortality and longer hospitalization.
GUSTO (Global Utilization of Streptokinase and Tissue Plasminogen Activator for Occluded Coronary Arteries)^66^	**Severe/life-threatening:** Intracranial bleeding or any bleeding with haemodynamic compromise requiring intervention. **Moderate:** Bleeding requiring transfusion but without haemodynamic instability. **Mild:** Any other overt bleeding not meeting above criteria.	Focuses on clinical outcomes such as need for transfusion and haemodynamic consequences rather than lab thresholds.	Severe GUSTO bleeds predict early in-hospital death and adverse cardiovascular outcomes.
ISTH (International Society of Thrombosis and Haemostasis)^62–64^	**Major:** Fatal bleeding, or symptomatic bleeding in a critical organ (intracranial, intraspinal, intraocular, pericardial, intra-articular, intramuscular with compartment syndrome), or causing ≥2 g/dL haemoglobin fall or ≥2-unit transfusion. **Clinically relevant non-major (CRNMB):** Bleeding not meeting major criteria but leading to hospitalization, medical/surgical intervention, or change/interruption of therapy.	Integrates both anatomic and functional impact of bleeding, bridging laboratory and clinical definitions.	Major and CRNMB bleeding are associated with higher morbidity and mortality in anticoagulated populations.
ACUITY (Acute Catheterization and Urgent Intervention Triage strategY)^65^	**Major:** Intracranial, retroperitoneal, intraocular bleeding, or any bleed causing ≥4 g/dL haemoglobin drop, or requiring ≥2-unit transfusion. **Minor:** Any observed bleeding with a smaller Hb fall (<4 g/dL).	Developed in the PCI/ACS setting to align bleeding events with procedural complications.	Major ACUITY bleeding independently predicts 1-year mortality and myocardial infarction.
BARC (Bleeding Academic Research Consortium)^6^	**Type 0:** No bleeding. **Type 1:** Minor, not actionable (e.g. self-resolving epistaxis). **Type 2:** Actionable bleeding requiring medical evaluation or therapy but not meeting criteria for major bleeding. **Type 3a:** Hb drop 3–5 g/dL or transfusion; **3b:** Hb drop ≥5 g/dL, cardiac tamponade, surgical control, or IV vasoactive agents; **3c:** Intracranial/intraspinal or intraocular with vision loss. **Type 4:** CABG-related bleeding (transfusion ≥5 units within 48 h or chest tube output ≥2 L/24 h). **Type 5:** Fatal (5a probable; 5b definite).	Combines anatomical, clinical, and laboratory elements into a unified scale applicable across trials.	Now endorsed by ESC, ACC, and FDA as the standard bleeding definition; correlates with mortality, MI, and therapy discontinuation.

BARC, Bleeding Academic Research Consortium; CABG, coronary artery bypass graft; GUSTO, Global Utilization of Streptokinase and Tissue Plasminogen Activator for Occluded Coronary Arteries; Hb, haemoglobin; ISTH, International Society on Thrombosis and Haemostasis; TIMI, Thrombolysis in Myocardial Infarction; ESC, European Society of Cardiology; ACC, American College of Cardiology; FDA, US Food and Drug Administration.

### Haemodynamic instability

Haemodynamic instability is one of the most immediate and clinically meaningful markers of severe bleeding. Hypotension, tachycardia, altered mentation, cool extremities, or rising lactate should trigger concern for occult or high-volume haemorrhage, independent of haemoglobin levels.^67^ Within the BARC classification, haemodynamic compromise requiring vasopressors or surgical intervention corresponds to type 3b, while fatal events are categorized as type 5.^6^

Early management is guided primarily by clinical presentation rather than laboratory thresholds. Initial priorities include airway protection, oxygen supplementation, rapid venous access, and administration of balanced crystalloids while avoiding excessive haemodilution.^68^ Continuous invasive arterial pressure monitoring provides a more reliable assessment of perfusion in unstable patients than non-invasive measurements.^67^

A structured, algorithm-based resuscitation strategy integrating timely volume replacement, targeted transfusion, and haemostatic measures is recommended in severe haemorrhage and facilitates early stabilization.^69^ Restrictive transfusion thresholds—generally 7–8 g/dL—are appropriate in most bleeding scenarios, except in cases of ongoing exsanguination or myocardial ischaemia.^70^ Specifically, in patients with acute MI, recent randomized evidence and meta-analyses suggest that a restrictive transfusion strategy may be associated with a signal towards worse ischaemic outcomes compared with more liberal approaches, warranting individualized transfusion thresholds in this setting.^71,72^

Persistently low blood pressure despite adequate fluid replacement identifies patients at highest short-term mortality risk and warrants early vasopressor support and expedited definitive bleeding control.

### Bleeding in critical anatomical sites

Bleeding in critical anatomical sites carries disproportionate prognostic weight and mandates prioritized evaluation.^73,74^ In critically ill anticoagulated patients, bleeding frequently occurs in the context of complex, multifactorial coagulopathies that require rapid pathophysiological assessment and coordinated management.^75^

ICH remains the most catastrophic anticoagulant-related event, with high early mortality and long-term disability, requiring immediate discontinuation of therapy, urgent neuroimaging, and drug-specific reversal when appropriate.^1^ GI bleeding is the most frequent critical-site event in patients on DOACs or DAPT and requires prompt stabilization, early PPI initiation, and urgent endoscopic assessment once safe.^51,76^ Other high-impact locations—including retroperitoneal, pericardial (including tamponade), airway, intra-articular, intraspinal, intraocular, and urinary tract bleeding—often deteriorate rapidly even with modest volume loss and require coordinated multidisciplinary management. Key implications and immediate priorities for each site are summarized in *Table 3*.

**Table 3:** Major critical-site bleeding: clinical features, pathophysiology, and immediate management priorities

Critical site bleed	Pathophysiologic rationale	Immediate management priorities
Intracranial (ICH, subdural, subarachnoid)	Even small haematomas can cause abrupt increases in intracranial pressure and irreversible neuronal injury. Mortality approaches 40%.	Immediate discontinuation of all antithrombotics; urgent neuroimaging (CT/MRI); specific reversal (idarucizumab for dabigatran, andexanet alfa for FXa inhibitors, 4-factor PCC + vitamin K for VKA); neurosurgical consultation for decompression when indicated.
Intraocular	Even minimal bleeding within the ocular chamber may result in acute visual loss due to retinal or vitreous haemorrhage; often spontaneous in anticoagulated elderly or hypertensive patients.	Immediate ophthalmologic evaluation; discontinue antithrombotic therapy; consider reversal only for sight-threatening or expanding haemorrhage; maintain head elevation and avoid increased venous pressure.
Pericardial (tamponade)	Even minor blood accumulation can severely impair diastolic filling and cardiac output; frequently iatrogenic during catheter-based interventions.	Urgent pericardiocentesis under echocardiographic guidance; volume resuscitation; reversal of anticoagulation; temporary pacing if bradycardic collapse.
Gastrointestinal	Erosive, ulcerative, or variceal lesions are amplified by impaired haemostasis and remain the leading preventable cause of fatal bleeding on OAC or DAPT.	Haemodynamic stabilization; high-dose IV PPI; urgent endoscopy with haemostatic intervention; targeted reversal when indicated.
Retroperitoneal/intra-abdominal	Hidden bleeding may lead to delayed recognition and progressive hypovolemia or abdominal compartment syndrome.	Contrast-enhanced CT for diagnosis; resuscitation; reversal of anticoagulation; transcatheter arterial embolization or surgery if active bleeding persists.
Airway/pulmonary (epistaxis, haemoptysis, post-intubation)	Risk of airway obstruction and asphyxia; bleeding often originates from mucosal trauma or bronchial vasculature.	Airway protection; topical or endoscopic haemostasis; nebulized tranexamic acid; interventional radiology or surgical management for refractory bleeding.
Genitourinary (gross haematuria, post-procedural)	Usually follows instrumentation or local trauma; may cause clot retention and urinary obstruction.	Temporary suspension of antithrombotic therapy; bladder irrigation or cystoclysis; urologic evaluation; reversal if ongoing major bleeding.
Musculoskeletal/intra-articular	Haemarthrosis produces local pressure and functional limitation; rarely life-threatening but clinically relevant.	Joint rest, compression, analgesia; aspiration if tense effusion; temporary interruption of anticoagulant therapy.

CT, computed tomography; MRI, magnetic resonance imaging; FXa, factor Xa; VKA, vitamin K antagonist; IV, intravenous; PPI, proton-pump inhibitor; OAC, oral anticoagulant; DAPT, dual antiplatelet therapy; ICH, intracranial haemorrhage; PCC, prothrombin complex concentrate.

### Laboratory measures

Laboratory evaluation complements clinical assessment and supports decisions regarding reversal. In VKA-treated patients, INR remains the reference marker guiding prothrombin complex concentrate (PCC)-based reversal.^77^ For DOACs, standard PT/aPTT provide qualitative information, whereas drug-specific assays (dilute thrombin time and ecarin-based assays for dabigatran, chromogenic anti-FXa assays for apixaban, betrixaban, rivaroxaban, or edoxaban), offer more accurate quantification to inform reversal strategy.^78^ When drug-specific assays are unavailable, standard coagulation tests may still provide indirect information. In particular, a normal thrombin time reliably excludes clinically relevant dabigatran levels, whereas a prolonged prothrombin time may indicate residual exposure to factor Xa inhibitors, especially rivaroxaban or edoxaban.^79^ Viscoelastic testing including thromboelastography (TEG) and rotational thromboelastometry (ROTEM) may assist in transfusion decisions during complex coagulopathy, though DOAC-specific thresholds remain unvalidated.^79^ A summary of laboratory tools and their interpretation is provided in *Table 4*.

**Table 4:** Laboratory assessment in anticoagulated patients with clinically relevant bleeding or requiring urgent procedures

Antithrombotic	First-line test(s)	Interpretation for rapid triage	Specialized assays (when available)	Key considerations/caveats
Vitamin K antagonists	PT/INR	INR reflects anticoagulant intensity and guides reversal decisions	—	In DIC or liver dysfunction, INR may be unreliable → supplement with viscoelastic or factor-based testing
Unfractionated heparin (UFH)	aPTT	aPTT ↑ = anticoagulant effect present	Anti-FXa (UFH-calibrated) if aPTT unreliable (e.g. lupus anticoagulant, low fibrinogen)	Protamine fully reverses UFH; results affected by hypofibrinogenemia and liver failure
Low-molecular-weight heparin (LMWH)	—	PT/aPTT not reliable for LMWH	Anti-FXa (LMWH-calibrated) for quantification in renal impairment, pregnancy, obesity, or major bleeding	Protamine provides partial reversal; peak levels ∼4 h after last dose
Fondaparinux	—	PT/aPTT not informative	Anti-FXa (fondaparinux-calibrated) if available	No specific antidote; rFVIIa/aPCC may be considered in refractory cases
Dabigatran	TT ± aPTT	**Normal TT** → excludes relevant levels; **prolonged aPTT** → suggests on- or above-therapy exposure; **normal aPTT** does *not* exclude	Dilute TT, Ecarin Clotting Time (ECT), or Ecarin Chromogenic Assay (ECA) for quantification; LC–MS/MS as gold standard	For life-threatening bleeding, SSC-ISTH suggests reversal if > 50 ng/mL; for high-risk procedures > 30 ng/mL
Rivaroxaban/Edoxaban/Betrixaban	PT	**Prolonged PT** → consistent with on/above-therapy levels; **normal PT** → does *not* exclude (reagent-dependent)	Chromogenic anti-FXa assay calibrated with the specific drug; LMWH/UFH-calibrated assays may exclude, not quantify	Strong reagent variability; always note last dose and renal function
Apixaban	PT, aPTT	Usually normal even at therapeutic levels	Drug-specific anti-FXa chromogenic assay for quantification	PT/aPTT are insensitive; normal values do *not* exclude clinically relevant concentrations
Bivalirudin/Argatroban	aPTT (or ACT in procedure)	aPTT ↑ expected; ACT used intra-procedurally	dTT or ECT if available	Short half-life; rapid decline after discontinuation if organ function preserved

***Operational note***: Quantitative testing (dTT, ECT, or ECA for dabigatran; drug-specific chromogenic anti-FXa for FXa inhibitors) should guide reversal or procedural timing. Reversal is generally considered for DOAC levels >50 ng/mL in major bleeding or >30 ng/mL before high-risk procedures, accounting for drug type, last dose timing, and renal or hepatic function.

aPTT, activated partial thromboplastin time; ACT, activated clotting time; aPCC, activated prothrombin complex concentrate; anti-FXa, anti–factor Xa assay; DIC, disseminated intravascular coagulation; DOAC, direct oral anticoagulant; DTI, direct thrombin inhibitor; dTT, dilute thrombin time; ECA, ecarin chromogenic assay; ECT, ecarin clotting time; INR, international normalized ratio; LC–MS/MS, liquid chromatography–tandem mass spectrometry; LMWH, low-molecular-weight heparin; PCC, prothrombin complex concentrate; PT, prothrombin time; rFVIIa, recombinant factor VIIa; SSC-ISTH, Scientific and Standardization Committee of the International Society on Thrombosis and Haemostasis; TT, thrombin time; UFH, unfractionated heparin.

## Management of major bleeding

### Supportive care

Supportive care represents the mainstay of initial management in major bleeding and must be initiated immediately and in parallel with definitive haemostasis. All antithrombotic agents, including anticoagulants, antiplatelets, and NSAIDs, should be discontinued promptly, and airway protection, oxygen supplementation, and large-bore venous access secured without delay. Early assessment must document drug type, timing of the last dose, renal and hepatic function, and potential interactions, as these directly influence clearance and the choice of reversal strategy.^73^

Initial resuscitation relies on rapid administration of balanced crystalloids to restore intravascular volume and improve perfusion, avoiding excessive saline loading to prevent hyperchloremic acidosis. Colloids offer no mortality benefit and are not recommended.^80^ Persistent hypotension despite adequate fluid resuscitation indicates evolving haemorrhagic shock and requires early vasopressor support, preferably norepinephrine, to maintain organ perfusion.^68^ A structured, physiology-guided transfusion strategy is preferred. Haemoglobin levels of 7–8 g/dL are acceptable for most patients^70^; however, in the patients with acute MI, recent evidence suggests that a more liberal transfusion strategy may be warranted, and transfusion thresholds should be individualized rather than strictly restrictive.^71,72^

Platelet transfusion should aim to maintain counts above 50 × 10^9^/L in patients with ongoing major bleeding or requiring urgent invasive procedures, while fibrinogen replacement—using cryoprecipitate or fibrinogen concentrate—should target plasma levels >100–150 mg/dL to ensure adequate clot formation.^18,73,81^

In the setting of exsanguinating haemorrhage or haemorrhagic shock, massive transfusion protocols based on fixed ratios of red blood cells, plasma, and platelets (typically 1:1:1) may be employed, particularly in trauma or uncontrolled bleeding, whereas goal-directed transfusion strategies guided by viscoelastic testing (TEG or ROTEM) are preferred when available, allowing tailored administration of plasma, fibrinogen, and platelets while limiting unnecessary transfusion.^68,69,73^

Fresh frozen plasma may be considered in the presence of coagulation factor deficiency or dilutional coagulopathy, although its use is limited by volume load and slower correction of haemostasis compared with factor concentrates; recombinant activated factor VII (rFVIIa) should be reserved for refractory, life-threatening bleeding when conventional supportive measures fail, given its thrombotic risk profile.^18,82,83^

Tranexamic acid improves survival in trauma-related haemorrhage when administered within 3 h of onset and should be considered early in appropriate patients.^84^ High-dose IV PPI therapy is recommended in suspected upper GI bleeding to stabilize clot formation and reduce early rebleeding risk.^85^

Special situations require tailored approaches. In severe renal impairment, dabigatran clearance is markedly prolonged and haemodialysis may be considered; in contrast, FXa inhibitors are minimally dialyzable. Uraemic platelet dysfunction may be corrected with desmopressin or cryoprecipitate.^86^ In hepatic dysfunction, traditional coagulation tests may be misleading, and viscoelastic monitoring is preferred for individualized factor replacement.^87^

Supportive care should therefore follow a systematic, algorithm-driven pathway integrating rapid stabilization, correction of coagulopathy, and early involvement of interventional radiology, surgery, or endoscopy for definitive source control.

### Specific reversals of anticoagulant agents

Rapid reversal should start immediately once anticoagulant therapy is discontinued, alongside haemodynamic stabilization and definitive haemostasis. Identifying the drug, dose, timing of last intake, and renal/hepatic function is crucial, as these variables dictate the choice and expected effectiveness of reversal. The goal is prompt haemostasis with minimal thrombotic rebound, consistent with contemporary multidisciplinary recommendations.^88,89^

*Table 5* outlines agent-specific mechanisms, available reversal strategies, dosing, and practical considerations.

**Table 5:** Specific reversal agents for antiplatelet and anticoagulant therapies

Agent	Mechanism	Specific reversal	Dosing/Onset	Clinical considerations
UFH	Antithrombin activation	Protamine sulfate	1 mg per 100 IU UFH (max 50 mg)	Immediate neutralization; caution for hypotension/anaphylaxis
LMWH	Anti-Xa activity	Protamine (partial)	1 mg per 1 mg enoxaparin (≤8 h)	∼60% reversal of anti-Xa effect
Fondaparinux	Indirect FXa inhibition	None specific (consider aPCC/rFVIIa)	—	Reserved for life-threatening bleeding
VKA	Inhibition of VK-dependent factors	Vitamin K + 4F-PCC	Vit K 5–10 mg IV + 25–50 IU/kg PCC	PCC preferred over plasma; rapid INR correction
Dabigatran	Direct thrombin inhibition	Idarucizumab	5 g IV (2 × 2.5 g) → onset <5 min	Complete reversal; safe profile
FXa inhibitors (apixaban, rivaroxaban, edoxaban)	Direct FXa inhibition	Andexanet alfa	Bolus + infusion per agent/dose	92% reduction in anti-FXa activity; thrombosis 10–14%
Multiple DOACs/heparins	Variable	Ciraparantag (PER977)	Single 100–300 mg IV bolus → effect in 30 min	Pan-specific; under phase 3 evaluation

DOAC, direct oral anticoagulant; UFH, unfractionated heparin; LMWH, low molecular weight heparin; PCC, prothrombin complex concentrate; 4F-PCC, four-factor PCC; aPCC, activated PCC; rFVIIa, recombinant factor VIIa; COX, cyclooxygenase; FXa, factor Xa; VK, vitamin K; DDAVP, desmopressin; GPI, glycoprotein IIb/IIIa inhibitor; IV, intravenous.

Anticoagulant-related bleeding requires a drug-specific and time-sensitive approach, integrating pharmacokinetic properties, severity of haemorrhage, and patient-specific factors. In the setting of major or life-threatening bleeding, immediate discontinuation of the anticoagulant and initiation of supportive measures are mandatory, followed, when indicated, by targeted reversal strategies aimed at rapidly restoring haemostasis while minimizing thrombotic risk.

Bleeding associated with unfractionated heparin (UFH) can be effectively reversed with protamine sulphate, administered at a dose of 1 mg per 100 units of heparin given within the previous two hours, up to a maximum of 50 mg, with slow infusion recommended to reduce the risk of hypotension or anaphylactoid reactions.^90^ In patients treated with LMWH, protamine provides only partial neutralization of anti-Xa activity—approximately 60–80%—with dosing guided by the timing and amount of the last administered dose; additional protamine may be considered in the presence of ongoing bleeding.^91^

In the absence of a specific antidote for fondaparinux, reversal options are limited. In life-threatening haemorrhage, rescue strategies with recombinant activated factor VII (rFVIIa) or activated prothrombin complex concentrate (aPCC) have been proposed, although supporting evidence is derived primarily from experimental and small observational studies.^92,93^

Bleeding complications related to VKAs require prompt correction of both anticoagulant effect and coagulation factor deficiency. In urgent or life-threatening situations, the combination of intravenous vitamin K (5–10 mg) and four-factor prothrombin complex concentrate (4F-PCC, 25–50 IU/kg) represents the preferred strategy, achieving rapid INR normalization and more reliable haemostatic correction than fresh frozen plasma.^77,94,95^ Plasma transfusion remains an alternative when PCCs are unavailable but is limited by slower onset, large infusion volumes, and transfusion-related complications.^96,97^ Isolated administration of vitamin K, either orally or intravenously, results in delayed and often incomplete reversal and should be reserved for non-urgent scenarios.^98,99^

Among DOACs, management strategies differ according to the mechanism of action. Dabigatran, a direct thrombin inhibitor, can be rapidly and specifically reversed with idarucizumab (5 g IV as 2 × 2.5 g boluses), which binds dabigatran with high affinity and achieves immediate and sustained normalization of coagulation parameters, with high rates of effective haemostasis in real-world practice.^100^ In patients with severe renal impairment, adjunctive haemodialysis may further enhance drug removal given the renal clearance profile of dabigatran.^101^

Bleeding associated with factor Xa inhibitors (apixaban, rivaroxaban, edoxaban, and betrixaban) may be managed with targeted or non-specific reversal strategies depending on drug availability, bleeding severity, and institutional protocols. Andexanet alfa, a recombinant inactive factor Xa decoy, was developed to provide rapid suppression of anti-Xa activity. In the ANNEXA-4 study, effective haemostasis was achieved in a majority of patients with major bleeding, although thrombotic events were observed, particularly among those in whom anticoagulation was not promptly resumed.^102^ However, the clinical role of andexanet alfa has become increasingly limited. Following withdrawal from the US Food and Drugs Administration (FDA) regulatory pathway, the agent is no longer commercially available in the USA^103^, while it currently remains approved in Europe.^104^ Importantly, this withdrawal was driven by commercial and regulatory considerations rather than new safety signals, although it has raised concerns regarding global availability and long-term implementation.

In addition, the lack of randomized comparative data between andexanet alfa and four-factor prothrombin complex concentrate (4F-PCC), signals of thrombotic complications, and logistical constraints have led many centres to preferentially adopt PCC-based reversal strategies in routine practice. Accordingly, when andexanet alfa is unavailable or institutional protocols favour alternative approaches, 4F-PCC represents an acceptable and widely used option, supported by observational data showing satisfactory haemostatic efficacy.^105^

Overall, contemporary management of factor Xa inhibitor–related bleeding increasingly relies on a pragmatic, multidisciplinary approach integrating non-specific reversal strategies, rapid source control, and early reassessment for anticoagulation resumption, while the role of andexanet alfa remains limited, context-dependent, and largely restricted to selected settings with local expertise. Emerging universal reversal agents, such as ciraparantag (PER977), offer the potential to neutralize multiple anticoagulant classes through non-covalent binding mechanisms. Early-phase studies have demonstrated rapid (<10 min) and sustained reversal of multiple anticoagulants (DOACs, heparins, fondaparinux) with an acceptable safety profile, although confirmatory data in patients with active bleeding or requiring urgent procedures are still awaited.^106–108^

The choice of reversal strategy should therefore be individualized, balancing the urgency of haemostasis against thrombotic risk, drug availability, and institutional protocols, ideally within a multidisciplinary framework involving cardiology, haematology, emergency medicine, and critical care.

### Interventional treatments

Interventional management follows a stepwise, time-sensitive pathway in which local haemostasis, correction of coagulopathy, and definitive source control proceed simultaneously. Immediate external control—firm compression or a proximal tourniquet when appropriate—remains the most effective first measure and is central to modern major bleeding algorithms.^109^

In ICH, management integrates neurological status, haematoma characteristics, and reversibility of anticoagulation. Surgical evacuation is considered only in selected cases (e.g. large supratentorial haematomas with mass effect or clinical deterioration) according to contemporary neurocritical care guidance.^74^

For GI bleeding, endoscopy is the preferred first-line approach, and the British Society of Gastroenterology/European Society of Gastrointestinal Endoscopy (BSG/ESGE) guideline emphasizes that procedures should not be delayed only because of OAC exposure. Aspirin is typically continued, while P2Y_12_ inhibitors and DOACs are managed based on procedural risk and haemostasis needs.^110^ When endoscopy is unfeasible or bleeding recurs, transcatheter arterial embolization offers high success rates. Modern embolic agents, such as n-butyl cyanoacrylate or coils, achieve excellent technical and clinical outcomes.^111^

When urgent surgery is required, definitive source control should not be postponed. In patients receiving DOACs, heparin bridging is generally unnecessary; short, structured interruption intervals (≈24–48 h depending on renal function and procedural risk) minimize perioperative thrombotic and bleeding complications.^112^

In epistaxis, first-line measures include external compression and topical vasoconstrictors, followed, if required, by resorbable nasal packing. This technique is preferred in patients on anticoagulant or antiplatelet therapy to reduce mucosal trauma and allow safer haemostasis, as recommended in the American Academy of Otolaryngology–Head and Neck Surgery Foundation (AAO-HNSF) guideline.^113^

Across all anatomic sites, the overarching principle is consistent: deploy the simplest effective local measure early, escalate to endoscopic, endovascular, or surgical treatment as needed, and individualize antithrombotic interruption and reversal to avoid unnecessary thrombotic exposure.

### Restarting anticoagulation after a bleeding event

Restarting anticoagulation after a major bleeding event requires a careful balance between thrombotic risk and the likelihood of recurrent bleeding. Once haemostasis is secured and the bleeding source definitively treated, prolonged interruption should be avoided, as withholding anticoagulation is consistently associated with higher rates of MI, ischaemic stroke, and mortality.^114^

Patients at high thrombotic risk, mechanical valves, recent VTE (<3 months), or AF with recent stroke/TIA or CHA_2_DS_2_-VASc ≥5 generally benefit from early resumption. When full anticoagulation is temporarily unsafe, short-acting parenteral prophylaxis (UFH/LMWH) may be used; intravenous UFH is preferred when rapid reversibility is required.^82^

Across DOAC-related bleeds, thrombotic events cluster in the first month and are substantially less common in patients who restart therapy.^115,116^ After GI bleeding, both DOAC and warfarin data support resumption once clinically stable.^117^ After ICH, evidence suggests a net clinical benefit for restarting in selected patients once neuroimaging stabilizes and the underlying lesion is secured.^118,119^

Beyond timing, the intensity and regimen of anticoagulation resumption have emerged as key determinants of bleeding recurrence without compromising thromboembolic protection.

Recent randomized trials have provided important insights into dose optimization and drug selection in high-risk settings.

In the RENOVE trial, patients with venous thromboembolism at high risk of recurrence requiring extended anticoagulation were randomized to reduced-dose vs. full-dose direct oral anticoagulants. Although non-inferiority for recurrent VTE was not formally met, reduced-dose therapy was associated with a substantial reduction in major or clinically relevant bleeding, with low absolute recurrence rates in both groups, supporting dose de-escalation as a potential bleeding-mitigation strategy in selected patients.^120^

Further support comes from comparative effectiveness studies evaluating DOAC selection after bleeding. The COBBRA study demonstrated a significantly lower risk of major bleeding with apixaban compared with rivaroxaban in patients treated for acute venous thromboembolism, reinforcing the relevance of agent-specific bleeding profiles when anticoagulation is resumed after a haemorrhagic event.^121^

In oncologic populations, the API-CAT trial further refined this paradigm by showing that reduced-dose apixaban was non-inferior to full-dose therapy for preventing recurrent cancer-associated thrombosis while significantly lowering clinically relevant bleeding, highlighting the role of dose tailoring in patients with intrinsically elevated bleeding risk.^122^

Collectively, these data support a more individualized approach to anticoagulation resumption after bleeding, integrating not only timing but also drug choice and dose intensity to optimize the balance between thromboembolic protection and bleeding risk.

When anticoagulation is temporarily impossible despite high thrombotic risk, non-pharmacologic strategies may be considered in carefully selected patients.

Among these, percutaneous left atrial appendage occlusion (LAAO) has emerged as the most extensively investigated alternative to long-term oral anticoagulation in patients with atrial fibrillation, particularly in those with a high bleeding burden or recurrent haemorrhagic complications, while temporary inferior vena cava filters may be considered in selected non-cardioembolic settings.^123,124^

More recently, outcome-driven randomized data have further expanded the evidence base supporting this approach. In the OPTION trial, conducted in patients with AF at elevated thromboembolic risk undergoing catheter ablation, LAAO with the WATCHMAN FLX device was associated with a significant reduction in non–procedure-related major or clinically relevant non-major bleeding compared with continued OAC, while remaining non-inferior for the composite endpoint of death, stroke, or systemic embolism over 36 months.^125^

Additional evidence derives from randomized and ongoing trials focusing on patients in whom long-term OAC is contraindicated or poorly tolerated. The COMPARE-LAAO trial is evaluating LAAO vs. antiplatelet therapy or no antithrombotic treatment in patients with AF and a formal contraindication to oral anticoagulation, addressing a clinically relevant population frequently encountered after major bleeding events.^126^ In parallel, the Occlusion-AF trial is assessing the non-inferiority of LAAO compared with DOACs in patients with AF and recent ischaemic stroke or transient ischaemic attack, using a composite endpoint that integrates both thromboembolic and major bleeding outcomes.^127^

Collectively, these data support a nuanced role for LAAO as a potential alternative strategy in selected patients at high thrombotic risk in whom anticoagulation cannot be safely maintained. However, current evidence also underscores that LAAO should not be viewed as a universal substitute for anticoagulation, but rather as a patient-specific option requiring multidisciplinary evaluation, careful anatomical assessment, and consideration of procedural risks. Accordingly, contemporary guidelines continue to reserve LAAO primarily for selected patients with clear contraindications to long-term OAC, pending further results from ongoing comparative trials.^46^

Ultimately, safe reinitiation of OAC requires targeted medication review (drug–drug interactions, especially with DOACs) and reassessment of antiplatelet therapy, avoiding unnecessary aspirin or DAPT.^128,129^ Structured patient counselling on recurrent bleeding symptoms remains essential.

## Management of minor bleeding

Minor bleeding events, such as epistaxis, gingival bleeding, superficial bruising, or self-limiting GI oozing, are frequent in patients receiving OACs or antiplatelet therapy and seldom require hospitalization or reversal.^73,130,131^ Management is primarily conservative and aimed at symptom control, correction of reversible factors, and avoidance of unnecessary treatment interruption.^77,114,132^

Initial steps include local haemostatic measures and assessment of contributing triggers such as mucosal trauma, uncontrolled hypertension, concomitant use of NSAIDs, SSRIs, oral steroids, or unnecessary aspirin, all of which significantly increase bleeding risk.^48–50^ Temporary withholding or delaying the next anticoagulant dose is usually sufficient until haemostasis is achieved. In VKA-treated patients, INR measurement is essential; if supratherapeutic, low-dose vitamin K (1–2 mg orally or IV) may facilitate controlled correction without complete reversal.^77^ For DOAC users, the clinical impact of formal drug-level testing is generally unnecessary, but documentation of last dose, renal function, hepatic function, and interacting drugs is important for tailored management.^56,133^

Upper GI bleeding warrants prompt initiation of a proton pump inhibitor (PPI), which reduces the risk of recurrence and supports mucosal healing.^51,76^ By contrast, in lower GI bleeding, PPI have no proven benefit, and management should primarily focus on haemodynamic stabilization, correction of reversible risk factors, and timely colonoscopic evaluation to identify and treat the bleeding source.^134,135^

In patients receiving concomitant antiplatelet agents, the indication for dual therapy should be reassessed using a risk-based approach, recognizing that irreversible platelet inhibitors require several days for functional recovery, whereas ticagrelor has a faster offset. In minor head trauma with a normal initial computed tomography (CT) scan, the risk of delayed ICH is very low; outpatient management with structured patient education regarding red-flag symptoms is appropriate.^136^

Overall, minor bleeds should prompt targeted evaluation, patient reassurance, and correction of modifiable drivers, avoiding unnecessary discontinuation of lifesaving antithrombotic therapy.

## Practical flow-chart

**Figure 1:**
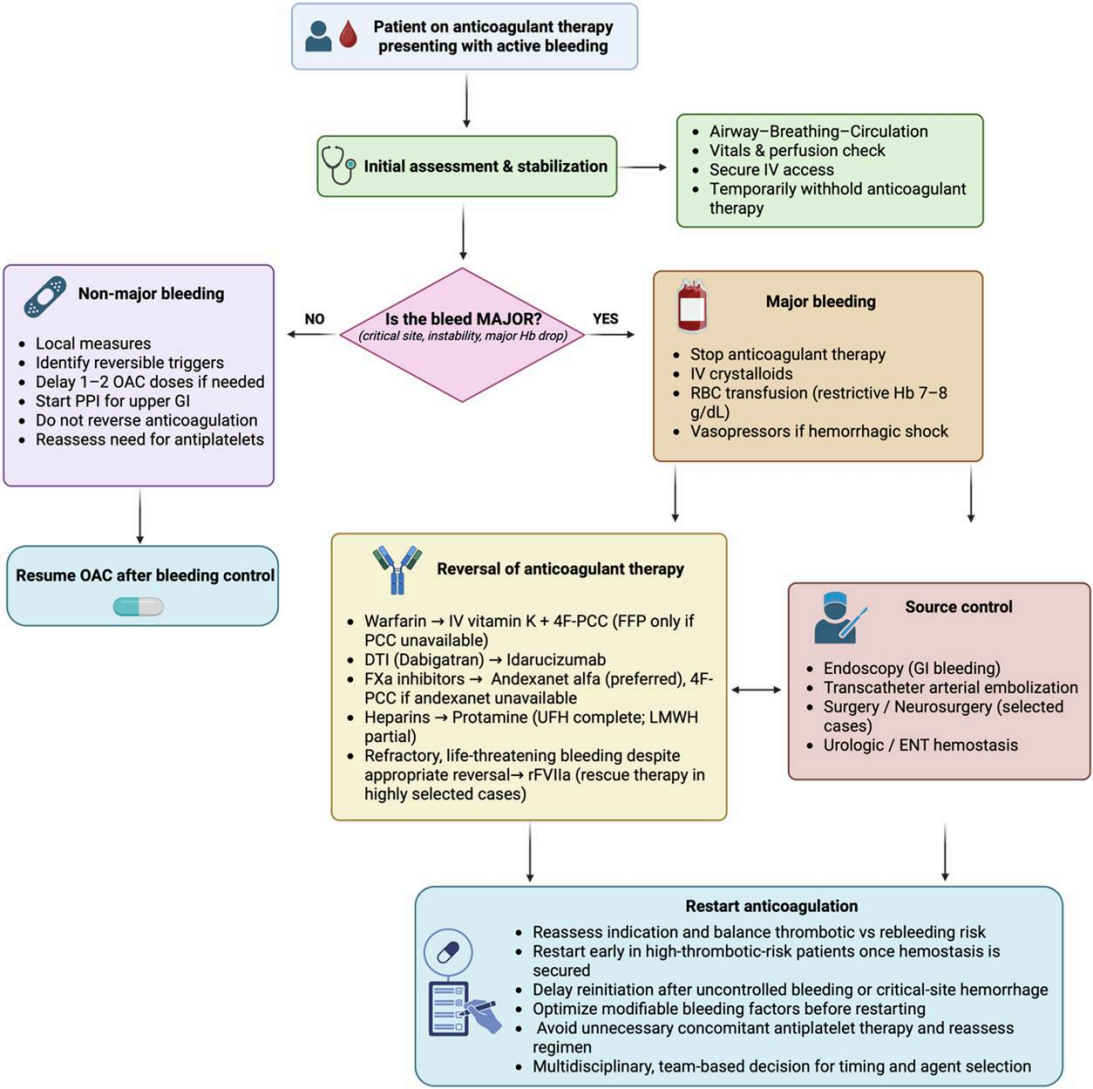
Pragmatic algorithm for the acute management of bleeding in patients receiving anticoagulant therapy. Abbreviations: OAC, oral anticoagulant; IV, intravenous; RBC, red blood cells; Hb, haemoglobin; GI, gastrointestinal; PPI, proton pump inhibitor; DTI, direct thrombin inhibitor; FXa, factor Xa; PCC, prothrombin complex concentrate; 4F-PCC, four-factor prothrombin complex concentrate; FFP, fresh frozen plasma; UFH, unfractionated heparin; LMWH, low-molecular-weight heparin; rFVIIa, recombinant factor VIIa; ENT, ear, nose, and throat.

*Figure 1* summarizes a pragmatic, risk-stratified approach to the management of bleeding in patients receiving oral anticoagulants, integrating early stabilization, bleeding severity assessment, targeted reversal strategies, and safe OAC resumption. The flow chart is designed to support real-world clinical decision-making across common scenarios, without replacing individualized clinical judgment.

After initial assessment and stabilization—including airway protection, circulatory support, and secure venous access—the pivotal step is the distinction between major and non-major bleeding, which directs subsequent management.

In non-major bleeding, conservative measures, identification and correction of reversible triggers, and temporary OAC dose adjustment or short-term interruption are generally sufficient, and routine pharmacological reversal is discouraged to minimize thrombotic risk.

Conversely, major bleeding mandates immediate OAC discontinuation, haemodynamic support, and early consideration of anticoagulant reversal. As outlined in the flow chart, reversal strategies prioritize drug-specific antidotes when available (idarucizumab for direct thrombin inhibitors), while the use of andexanet alfa for factor Xa inhibitor–related bleeding should be approached with caution and reserved for experienced centres. Four-factor prothrombin complex concentrate (4F-PCC) is recommended for VKA-related bleeding and represents a widely used alternative for factor Xa inhibitors, particularly when specific antidotes are unavailable or contraindicated. Recombinant activated factor VII (rFVIIa) should be restricted to exceptional, life-threatening cases refractory to appropriate reversal, given its limited evidence base and associated thrombotic risk. Concomitantly, prompt source control—endoscopic, interventional, or surgical—should be pursued whenever feasible.

Finally, the flow-chart underscores the importance of timely OAC resumption, which requires reassessment of the original indication and a careful balance between thromboembolic and rebleeding risk, favouring early restart in high-risk patients once haemostasis is secured and delayed reinitiation in the setting of uncontrolled or critical-site bleeding.

## Conclusions

Bleeding is increasingly recognized as a major determinant of prognosis in patients treated with anticoagulants. Accordingly, contemporary management has shifted from a purely thromboembolic focus towards structured bleeding prevention and standardized care pathways. Effective strategies include accurate bleeding-risk stratification, systematic review of modifiable contributors, and individualized antithrombotic selection. When bleeding occurs, rapid assessment, targeted reversal, and coordinated multidisciplinary management are essential to restore haemostasis. Equally important is the safe and timely reinitiation of anticoagulation, guided by patient-specific thrombotic and re-bleeding risks. Ultimately, a coordinated, patient-centred approach remains integral to optimizing outcomes in individuals receiving anticoagulant therapy.

## Data Availability

No new data were generated or analysed in support of this research.
